# The interrelationship among exercise intensity, endothelial function, and ghrelin in healthy humans

**DOI:** 10.14814/phy2.70213

**Published:** 2025-04-11

**Authors:** Kara C. Anderson, Emily E. Grammer, Benjamin Stephenson, Macy E. Stahl, Nathan R. Weeldreyer, Zhenqi Liu, Kaitlin M. Love, Jason D. Allen, Arthur Weltman

**Affiliations:** ^1^ Department of Kinesiology, School of Education and Human Development University of Virginia Charlottesville Virginia USA; ^2^ Department of Medicine University of Virginia Health System Charlottesville Virginia USA

**Keywords:** acute exercise, gut hormones, sex differences, vascular function

## Abstract

Ghrelin circulates in acylated (AG) and deacylated (DAG) isoforms and both may impact endothelial function (EF). Although acute exercise has been shown to modulate ghrelin levels and EF, data on the impact of exercise intensity on these parameters are scarce. To investigate the effect of exercise intensity and sex on EF and ghrelin levels, nine males (age: 43.8 ± 10.3 y; BMI: 22.5 ± 1.8 kg/m^2^) and eight females (age: 33.75 ± 10.2 y; BMI: 22.4 ± 1.6 kg/m^2^) completed a maximal cycle ergometer lactate threshold (LT)/VO_2peak_ test. This test determined the exercise intensity for three visits: (a) CON, no exercise; (b) MOD, the power output (PO) at LT; (c) HIGH, the PO associated with 75% of the difference between LT and VO_2peak_. Ghrelin levels and EF [flow‐mediated dilation (FMD), shear rate (SR)] were measured at baseline and then 30–120 min post‐exercise. HIGH and MOD increased FMD (*p* < 0.0001). Each ghrelin isoform was suppressed by HIGH; only females exhibited reduced DAG levels in HIGH compared to MOD and CON (*p* < 0.0001–0.004). FMD was associated with ghrelin levels in females (*r* = −0.26–0.47). High‐intensity exercise is key for ghrelin suppression and appears to only be weakly/moderately related to EF.

## 1 INTRODUCTION

Kojima and Kangawa discovered ghrelin as an endogenous ligand to the growth hormone secretagogue receptor 1a (GHSR1a) in 1999 (Kojima et al., 1999). First studied to evaluate its role in the stimulation of growth hormone (GH) release from the anterior pituitary gland, research on ghrelin expanded to examine how it affects the endocrine, cardiovascular, digestive, and immune systems (Müller et al., 2015).

Ghrelin circulates in two forms, acylated (AG) and deacylated (DAG), where the majority circulates as DAG (~78% of total ghrelin (TG)) (Müller et al., 2015). The less abundant AG (~22% of TG) binds to GHSR1a and is catalyzed by ghrelin O‐acyltransferase (GOAT). The precursor of this catalyzation is DAG (Müller et al., 2015; Tong et al., 2013), though this form binds to a receptor yet to be identified. Differentiating between the two forms is important as data suggests that AG and DAG can act independently, synergistically, or antagonistically within the body (Müller et al., 2015).

AG and DAG are involved in maintenance of vascular endothelial function in healthy individuals by optimizing the balance of the vasodilator nitric oxide (NO) and the vasoconstrictor endothelin‐1 (ET‐1), which is critical in mitigating cardiovascular disease risk. In certain disease states, however, this balance may be disrupted in favor of vasoconstriction (Verma & Anderson, 2002). DAG has been shown to increase NO production in porcine endothelial cells (Grossini et al., 2011), while in humans, an infusion of AG restored the balance of NO and ET‐1 in individuals with metabolic syndrome (Tesauro et al., 2009). Additionally, sex appears to impact both ghrelin levels and endothelial function (EF). Females have higher levels of ghrelin and its isoforms compared to males (Douglas et al., 2017). In young adults, there are reported sex differences in the flow‐mediated dilation (FMD, a non‐invasive method to measure EF) response to shear stress, with females showing preserved EF (Tremblay et al., 2019). Sex differences have also been reported in the rate of decline in both macro‐ and microvascular EF associated with aging (Moreau, 2019). As endothelial dysfunction precedes the development of atherosclerosis, identifying the role of sex and the interaction between ghrelin and EF should aid in the development of treatment strategies that target the endothelium.

Exercise improves vascular function in healthy individuals and in disease states (Green et al., 2004; Johnson et al., 2012; Pierce et al., 2010), and also decreases cardiovascular disease risk (Shephard & Balady, 1999). The exercise response may be mediated, in part, through the interaction between ghrelin and EF as exercise also affects AG and DAG levels (Anderson et al., 2021; Broom et al., 2009, 2017; Burns et al., 2007; Douglas et al., 2017). Acute exercise has been shown to alter EF; however, sex and differences in exercise intensity/dose have led to inconsistent results (Hallmark et al., 2014; Jones et al., 2010; Rognmo et al., 2008). In addition, chronic exercise training has been shown to attenuate age‐associated vascular dysfunction in males and in estrogen‐sufficient females, but not in estrogen‐deficient females, suggesting a complex interaction between vascular adaptations to exercise, aging, and sex (Black et al., 2009; Pierce et al., 2010).

Identifying the ideal exercise intensity/dose to optimize EF and elucidate the contributing factors (e.g., ghrelin release) associated with the effects of acute exercise is critical for designing intervention strategies to attenuate the development of overt cardiovascular disease. A recent meta‐analysis published by our group determined that exercise suppresses ghrelin levels and that exercise intensity moderates that relationship; however, most studies included in the analysis utilized a single moderate‐intensity exercise bout (Anderson et al., 2021). Although the meta‐analysis examined all isoforms of ghrelin, it lacked power to show any effects of ghrelin isoforms as the majority of studies only measured AG.

Therefore, the purpose of this study was to investigate the effects of acute exercise intensity on EF and all ghrelin isoforms in untrained males and females. We hypothesized that high intensity exercise would lead to the greatest suppression of ghrelin levels and the largest improvement in vascular function. We also hypothesized that females would have an augmented FMD response to acute exercise compared to males.

## 2 MATERIALS AND METHODS

### 2.1 Participants

Individuals between the ages of 18–55 years were recruited for this study. They were selected for screening if they were untrained, non‐smoking, and weight‐stable (<3 kg over 3 months) and had a healthy weight BMI between 18.5 and 24.9 kg/m^2^. Criteria for exclusion included a history of Type 2 Diabetes (T2D), pregnancy/fertility treatments, disorders of the endocrine and gastrointestinal systems, and/or any medications/treatments that effected the ability to safely exercise or measure hormones and vascular function. Individuals arrived at the University of Virginia Clinical Research Unit (CRU) between 7 and 9 am after an overnight fast for all visits. Subjects were asked to refrain from exercise and alcohol consumption for 24 h, and tobacco products and caffeine use for 12 h before each CRU admission. All females were premenopausal in the early follicular phase of their menstrual cycle. The study was conducted in accordance with the Declaration of Helsinki; the protocol was approved by the University of Virginia Institutional Review Board (IRB‐HSR # 200241), and all subjects provided written informed consent.

### 2.2 Screening period

Subjects' body fat percentage (BF%) was assessed via dual energy x‐ray absorptiometry (DEXA, Hologic Horizon). Peak oxygen consumption (VO_2peak_) and lactate threshold (LT) were determined via an incremental test on a cycle ergometer (Lode Model 960,900). Subjects began at an initial power output of 50 W and power output was increased by 25 W every 3 min until volitional fatigue. Indirect calorimetry using standard open circuit spirometry (Vyntus, Vyaire, Yorba Linda, CA) was used to measure oxygen consumption and carbon dioxide production (and to provide min‐by‐min kcal to equate caloric expenditure between the two exercise bouts). Blood from an indwelling catheter placed in an antecubital vein was sampled at the end of each stage and assayed for lactate (YSI Instruments 2900, Yellow Springs, OH, USA). The LT was determined as the power output just prior to the curvilinear increase in blood lactate and the VO_2_ at this power output was chosen as VO_2_ at LT. The highest 1 min segment VO_2_ attained was chosen as VO_2peak_.

### 2.3 Testing period

**FIGURE 1:**
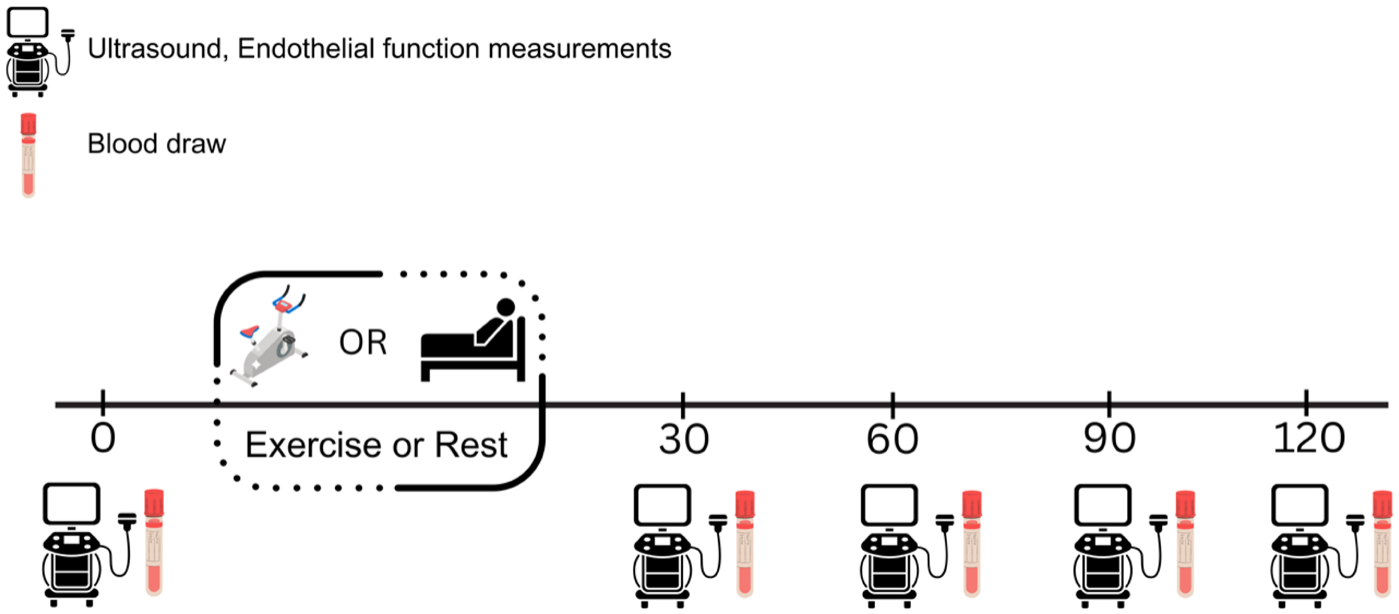
Testing Visit Outline.

The testing period consisted of three visits in random order: Control (CON, no exercise), moderate intensity exercise (MOD, power output at LT), and high‐intensity exercise (HIGH, power output associated with 75% of the difference between LT and peak). Caloric expenditure was matched within each subject for both exercise conditions (with a caloric goal between 200 and 300 calories), and participants exercised during each bout until that expenditure was reached. Testing visits are outlined in Figure 1. There was a minimum of 72 h between exercise sessions for males, and females completed their visits monthly to standardize data collection during the early follicular phase of their menstrual cycle. Participants recorded their diet the day before the first testing visit and were asked to replicate the same diet prior to each testing visit using a take‐home diet log.

Brachial artery FMD was measured at baseline (before exercise), 30, 60, 90, and 120 min post‐exercise. Participants were instructed to lay supine with right forearm extended (due to catheter placement in left arm). The location of the probe was marked during the first testing visit at baseline, and a measurement from the antecubital fold to the probe was recorded to allow for consistent probe placement for repeated measurements. A manual blood pressure cuff was placed distally from the antecubital fold and was inflated 50 mmHg above each participant's resting blood pressure for forearm occlusion. A high‐resolution ultrasound (Philips EPIC‐Q7) and a L12–5 linear transducer were used to obtain images at baseline and on r‐wave trigger for 2 min following cuff release to determine peak diameter. All images were analyzed via Brachial analyzer program (Medical Imaging Applications). Images were analyzed by two investigators (KCA and EEG) who were blinded to condition. The interclass correlation coefficient within our lab is 0.91. Baseline and peak diameter values were obtained for the FMD calculation. Post‐occlusion shear rate (SR) was calculated using the following formula: 4* (mean blood velocity/brachial artery diameter), where mean blood velocity is calculated by dividing the peak velocity in half (Thijssen et al., 2019). Peak velocities were automatically calculated by the ultrasound machine and recorded, and an angle of isonation of 60 degrees was used for all images.

### 2.4 Biochemical analyses

An indwelling catheter was inserted into the antecubital vein and blood was sampled at the same timepoints as FMD. Blood lactate was immediately analyzed (YSI Instruments 2900, Yellow Springs, OH, USA). Blood to measure TG, AG, and DAG was collected in 3 mL EDTA vacutainers containing 0.06 mL of protease inhibitor AEBSF and was centrifuged for 10 min at 3000 rpm at 4°C. 100 μL of hydrochloric acid 1 N was added to the plasma aliquots immediately after centrifugation. Plasma ghrelin was stored at −80°C for later analysis. Ghrelin was analyzed using Bertin Pharma ELISA kits by the University of Virginia Center for Research in Reproduction, Ligand Assay and Analysis Core. The intraassay variability for AG (RRID:AB_2936966) was 4.4% with a minimum detection limit of 3.0 pg/mL; for DAG (RRID:AB_2819343) the variability was 2.9% with a minimum detection limit of 2.0 pg/mL. All samples were run in duplicate.

## 3 STATISTICS

Data was analyzed via R (Version 2023.06.1+524). Based on previous literature (Douglas et al., 2017), assuming a power of 80% for an ANOVA with significance of *α* = 0.05, an adequate sample size of *n* = 8 per group was determined a priori to assess sex differences between acute exercise, FMD, and ghrelin levels. Power calculations were made with R Studio “pwr” package version 1.3. Baseline comparisons were evaluated using independent sample t‐tests, and normality was assessed using Q‐Q plots and the Shapiro–Wilk tests.

Several linear mixed models were used to examine the ghrelin and vascular response to exercise using R package “lmertest” (Version 3.1‐3). Based on the recommendations of Atkinson et al. and evidence that the traditional %FMD calculation violates statistical assumptions, our data fulfilled the requirements (where the slope of the linear regression model with the logged baseline diameter and logged peak diameter was <1) to logarithmically transform the values (Atkinson et al., 2009, 2013; Atkinson & Batterham, 2013). For the FMD model, the outcome was the difference between the logarithmically transformed values of baseline and post‐occlusion diameters (FMD_diff_), where time, sex, and condition (HIGH, MOD, CON) were fixed factors, and the logarithmically scaled baseline diameter and age were random factors. We also calculated an adjusted %FMD mean and standard error by backlogging the values from the logarithmically adjusted model with the following formulas:

$$\text{Mean}=\left[\left(e^{\text{Logged Mean}}-1\right)\times 100\right]$$

$$\text{Standard Error}=\left[\left(\left(e^{\text{Logged}\;\mathrm{SE}}-1\right)\times 100\right)\times \surd n\right]$$

For SR: time, sex, and condition were fixed factors, and baseline SR and age were random factors. For each ghrelin isoform: time, sex, and condition were fixed factors, and baseline value and age were random factors. We also calculated incremental area under the curve (AUC) for the following variables: FMD_diff_, SR, TG, AG, and DAG using the trapezoidal method in R package “auctime” (Version 2.0.0). For each AUC model, condition and time were fixed factors, and age was a random factor. For all models, Satterwhite's approximation was utilized to determine significance. F tests of nested models were used to determine differences in fixed effects. Estimated marginal means (EMM) were utilized to estimate the means that were adjusted for the factors in each model using R package “emmeans” (Version 1.8.9). Correlations were calculated using repeated measures correlations through the “rmcorr” R Package (Version 0.6.0), where correlations were calculated using subject as the grouping factor. Significance was set a priori as *p* < 0.05. Data from linear mixed models are reported as mean ± SE; data in tables are reported as mean ± SD.

## 4 RESULTS

Nine males (age: 43.8 ± 10.4 y; BMI: 22.5 ± 1.8 kg/m^2^; VO_2peak_: 35.2 ± 6.9 mL/kg/min) and eight females (age: 33.75 ± 10.2 y; BMI: 22.4 ± 1.6 kg/m^2^; VO_2peak_: 29.1 ± 4.5 mL/kg/min) completed the study. Sample characteristics are shown in Table 1. Females had significantly higher BF% (*p* = 0.0002), baseline TG (*p* = 0.04), and DAG (*p* = 0.0015), while males had a higher VO_2peak_ (*p* = 0.01–0.05) and baseline brachial artery diameter (*p* < 0.0001). Given that age between males and females approached statistical significance (*p* = 0.06), and based on data indicating age affects EF (Black et al., 2009) and ghrelin levels (Jones et al., 2010), we added age to all statistical models. Regarding exercise data, heart rate (HR), power output, and duration were significantly different between HIGH and MOD by design (*p* < 0.05). Males had a higher caloric expenditure and power output in both exercise conditions compared to females (*p* < 0.0001–0.05).

**TABLE 1:** Sample demographics.

	Males	Females	*p*‐Value
*N*	9	8	–
Age (years)	43.8 ± 10.4	33.75 ± 10.2	**0.06**
BMI (kg/m^2^)	22.5 ± 1.8	22.4 ± 1.6	0.92
Total BF (%)*	22.7 ± 4.6	33.7 ± 4.5	**0.0002**
VO_2peak_ (mL/kg/min)*	35.2 ± 6.9	29.1 ± 4.5	**0.05**
VO_2peak_ (L/min)*	2.3 ± 0.4	1.8 ± 0.4	**0.01**
Baseline AG (pg/mL)	90.25 ± 51.0	112.6 ± 54.4	0.17
Baseline DAG (pg/mL)*	93.3 ± 35.6	130.2 ± 39.2	**0.0015**
Baseline TG (pg/mL)*	186.2 ± 78.8	239.9 ± 81.0	**0.04**
AG:DAG	0.82 ± 0.29	0.94 ± 0.41	0.29
Baseline Artery Diameter (mm)*	4.15 ± 0.58	3.33 ± 0.33	**<0.0001**

Exercise Sessions	MOD	HIGH	MOD	HIGH
Duration (min)^a^	52:50 ± 8:27	29:54 ± 5:45	52:14 ± 06:04	35:40 ± 5:21
Energy Expenditure (kcal)	298.9 ± 30.75^b^	299.8 ± 30.75^b^	260.6 ± 35.8	258.4 ± 32.8
Average HR (bpm)^a^	124.1 ± 9.2	165.7 ± 11.8	134.3 ± 22.0	169.8 ± 20.4
Average Power Output (watts)^a^	71.5 ± 21.2^b^	140.0 ± 34.5^b^	65.1 ± 11.8	104.7 ± 14.2
%VO_2peak_ ^a^	65.4 ± 5.4^b^	88.6 ± 5.7	74.2 ± 6.5	93.3 ± 2.8

^*^ *p* < 0.05.

^a^ Difference between MOD and HIGH, *p* < 0.0001.

^b^ Difference within the same condition between males and females, *p* ≤ 0.05.

### 4.1 Endothelial function

**FIGURE 2:**
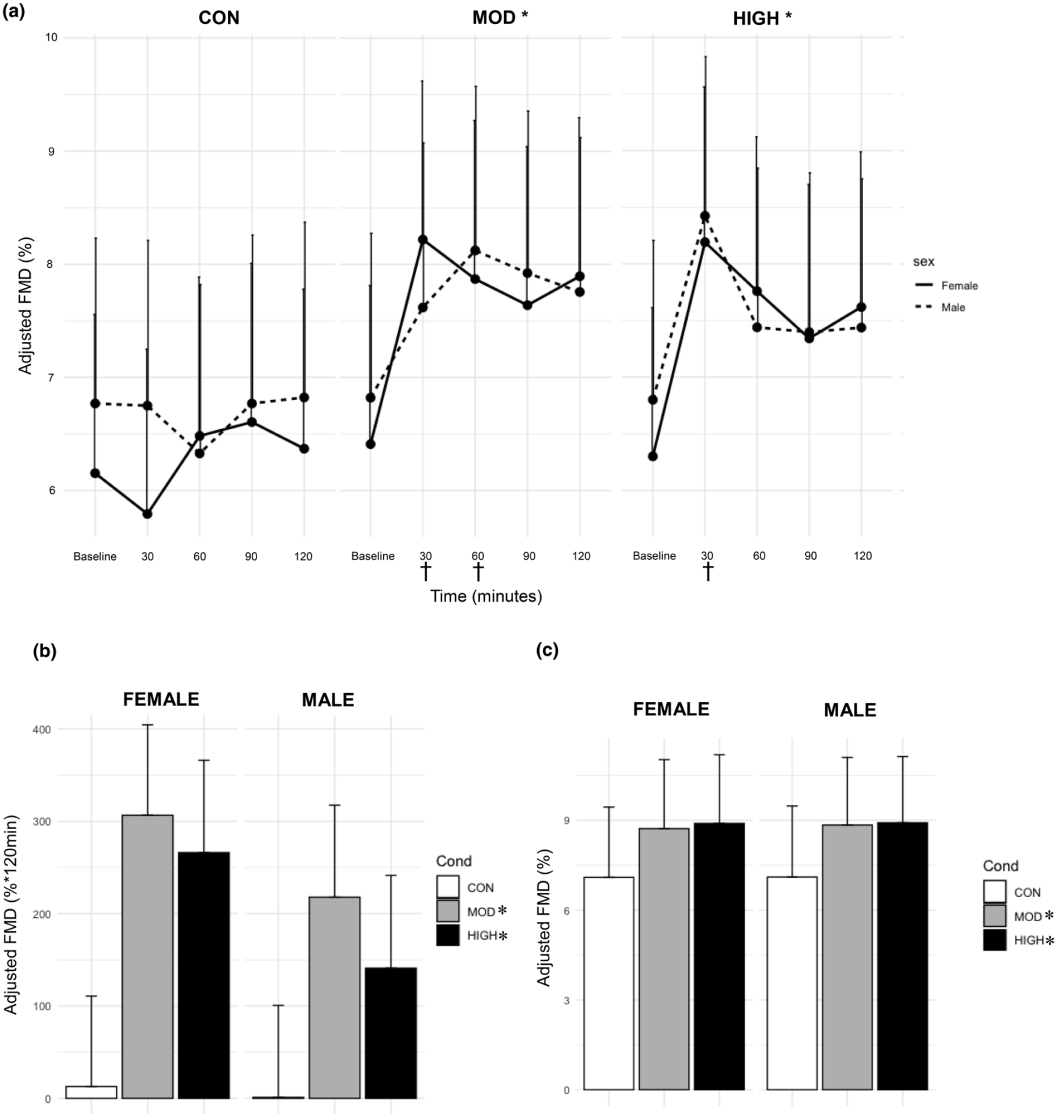
Effect of Condition on (a) Adjusted %FMD, (b) FMD Incremental AUC, and (c) Peak FMD. Data are from estimated marginal means of the linear mixed model and are presented as mean ± S.E. *; significantly different than CON, *p* ≤ 0.0002 †; significantly different from each condition's baseline value, *p* ≤ 0.02.

There was a significant main effect of condition (*p* < 0.0001, Figure 2a) and time (*p* < 0.0001), and an interaction between condition and time (*p* = 0.009). There was no effect of sex (*p* = 0.45). CON (Adjusted %FMD: 6.48 ± 1.53%) was significantly lower than MOD (7.46 ± 1.54%, *p* < 0.0001) and HIGH (7.62 ± 1.53%, *p* < 0.0001). There was a larger FMD response at 30 (7.50 ± 1.57%, *p* = 0.001), 60 (7.32 ± 1.57, *p* = 0.002), 90 (7.28 ± 1.58%; *p* = 0.006), and 120 min (7.32 ± 1.59%, *p* = 0.004) compared to baseline (6.45 ± 1.57%), regardless of condition. In addition, FMD in HIGH was significantly elevated at 30 min post‐exercise (8.30 ± 1.79%) compared to baseline (6.55 ± 1.81%, *p* = 0.0007), and MOD was significantly elevated 30 (7.91 ± 1.75%, *p* = 0.02) and 60 min post‐exercise (7.99 ± 1.75%, *p* = 0.009) compared to baseline (6.62 ± 1.74%). The FMD response at 30 min in HIGH was significantly larger than at 30 min in CON (6.27 ± 1.28, *p* < 0.0001). The FMD response at 30 and 60 min in MOD was significantly larger than at both of the same timepoints in CON (30: *p* = 0.0006; 60: 6.41 ± 1.28%, *p* = 0.001).

For the FMD_diff_ AUC model, there was a main effect of condition (*p* = 0.0001, Figure 2b), where CON (6.75 ± 98.40%*120 min) had significantly lower FMD than MOD (259.4 ± 98.39%*120 min, *p* = 0.0001) and HIGH (197.0 ± 95.51% *120 min, *p* = 0.002). There were no differences between MOD and HIGH (*p* = 0.76). There was no effect of sex (*p* = 0.32). Peak FMD revealed a significant effect of condition (*p* < 0.0001, Figure 2c), where CON (7.19 ± 1.88%) had a significantly lower peak than MOD (8.77 ± 1.88%, *p* = 0.0002) and HIGH (8.90 ± 1.78%, *p* = 0.0001). There were no differences between HIGH and MOD (*p* = 0.94).

**FIGURE 3:**
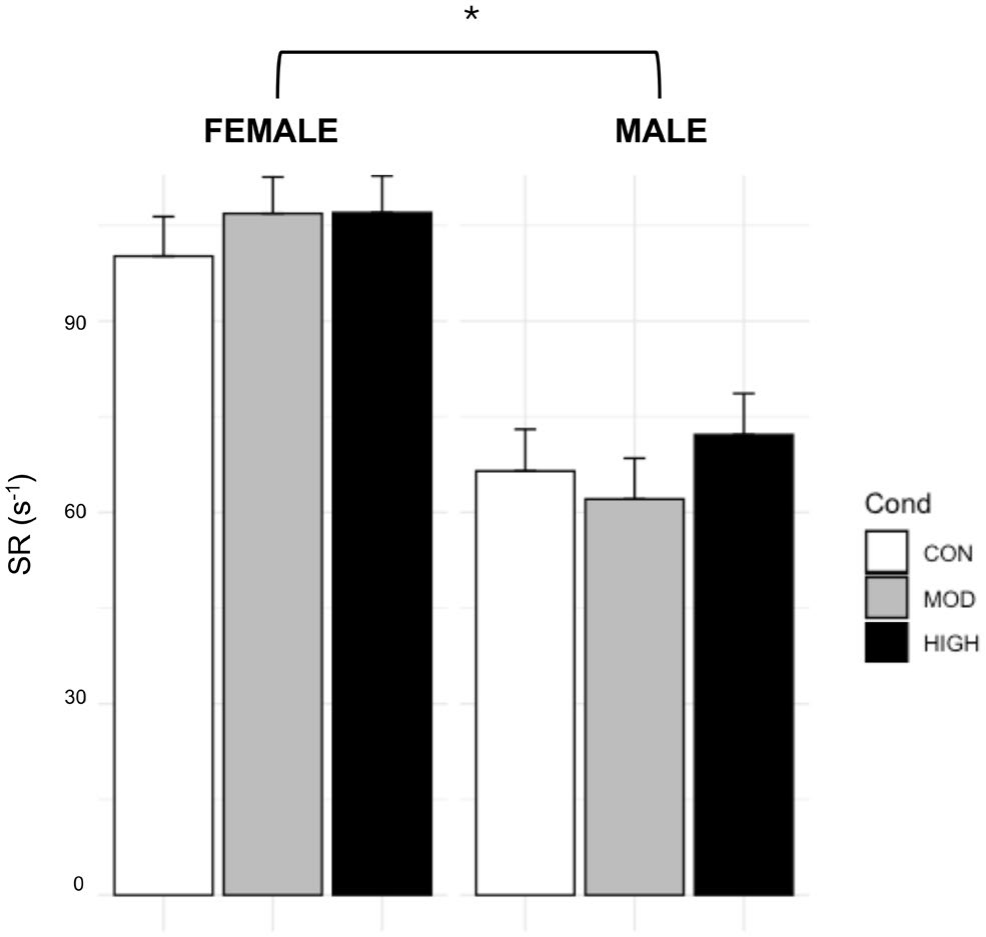
Effect of Condition on Shear Rate Timepoint Data. Data are from estimated marginal means of the linear mixed model and are presented as mean ± SE. *; significantly different between males and females, *p* < 0.0001.

For SR, there were significant main effects of sex (*p* < 0.0001, Figure 3), where females had elevated SR (104.7 ± 4.02 s^−1^) compared to males (66.9 ± 4.60 s^−1^) regardless of condition and when adjusted to baseline. There was no effect of condition or time (*p* = 0.48–0.52). There were no significant differences within the SR AUC model (not shown, *p* = 0.37–0.84).

### 4.2 Total ghrelin

**FIGURE 4:**
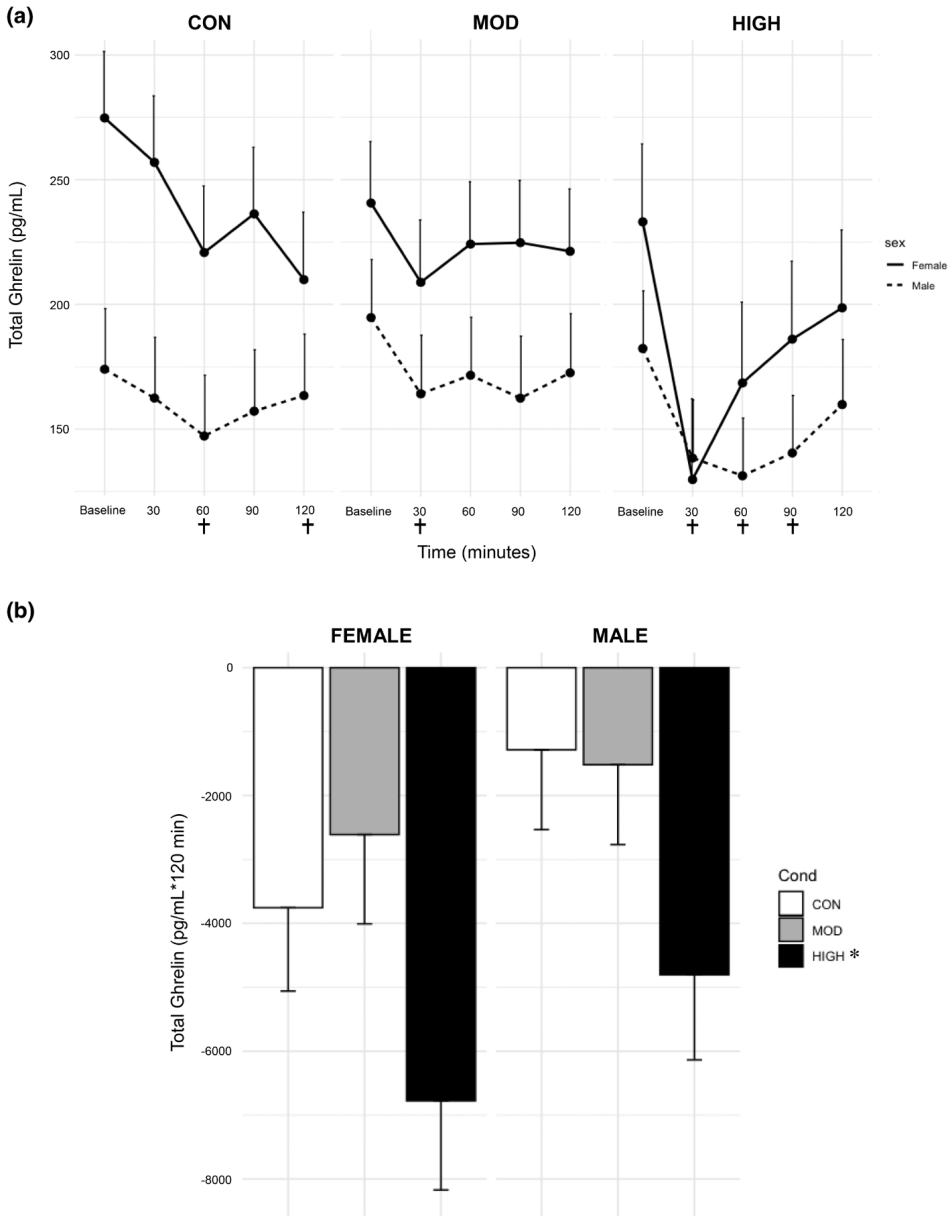
Effect of Condition on Total Ghrelin Levels with (a) Timepoint data and (b) Incremental AUC data. Data are from estimated marginal means of the linear mixed model and are presented as mean ± S.E. *; significantly different from CON and MOD *p* < 0.03; † significantly different from each condition's baseline value, *p* ≤ 0.04.

There were significant main effects for sex (*p* = 0.009) and time (*p* < 0.0001, Figure 4). There was also a significant interaction between condition and time (*p* = 0.009, Figure 4). Regardless of condition, TG levels were higher in females (216.0 ± 21.3 pg/mL) compared to males (161.0 ± 20.7 pg/mL). For the main effect of time, TG was decreased at all time points compared to baseline (*p* < 0.0001–0.001). TG levels were significantly decreased at 60 (184.0 ± 21.2 pg/mL, *p* = 0.004) and 120 min (187.0 ± 21.5 pg/mL, *p* = 0.02) compared to baseline in CON (224.0 ± 21.2 pg/mL). TG levels were significantly decreased at 30 min (186.0 ± 20.2 pg/mL, *p* = 0.04) compared to baseline in MOD (218.0 ± 21.0 pg/mL). Levels were significantly decreased at 30 (134.0 ± 22.5 pg/mL, *p* < 0.001), 60 (150.0 ± 22.5 pg/mL, *p* = 0.0001), and 90 min (163.0 ± 22.1 pg/mL, *p* = 0.004) compared to baseline in HIGH (208.0 ± 22.1 pg/mL, *p* < 0.0001–0.004). TG was significantly lower at 30 (*p* = 0.007) and 60 min (*p* = 0.03) in HIGH compared to the same timepoints in MOD, and lower at 30 min compared to the same timepoint in CON (210 ± 21.2 pg/mL, *p* = 0.006).

For the AUC model, there was a significant effect of condition (*p* = 0.01, Figure 4b), where HIGH (−5791 ± 974 pg/mL *120 min) was significantly lower than CON (−2520 ± 918 pg/mL *120 min, *p* = 0.03) and MOD (−2064 ± 955 pg/mL *120 min, *p* = 0.01). There were no differences between males and females (*p* = 0.10).

### 4.3 Acylated ghrelin

**FIGURE 5:**
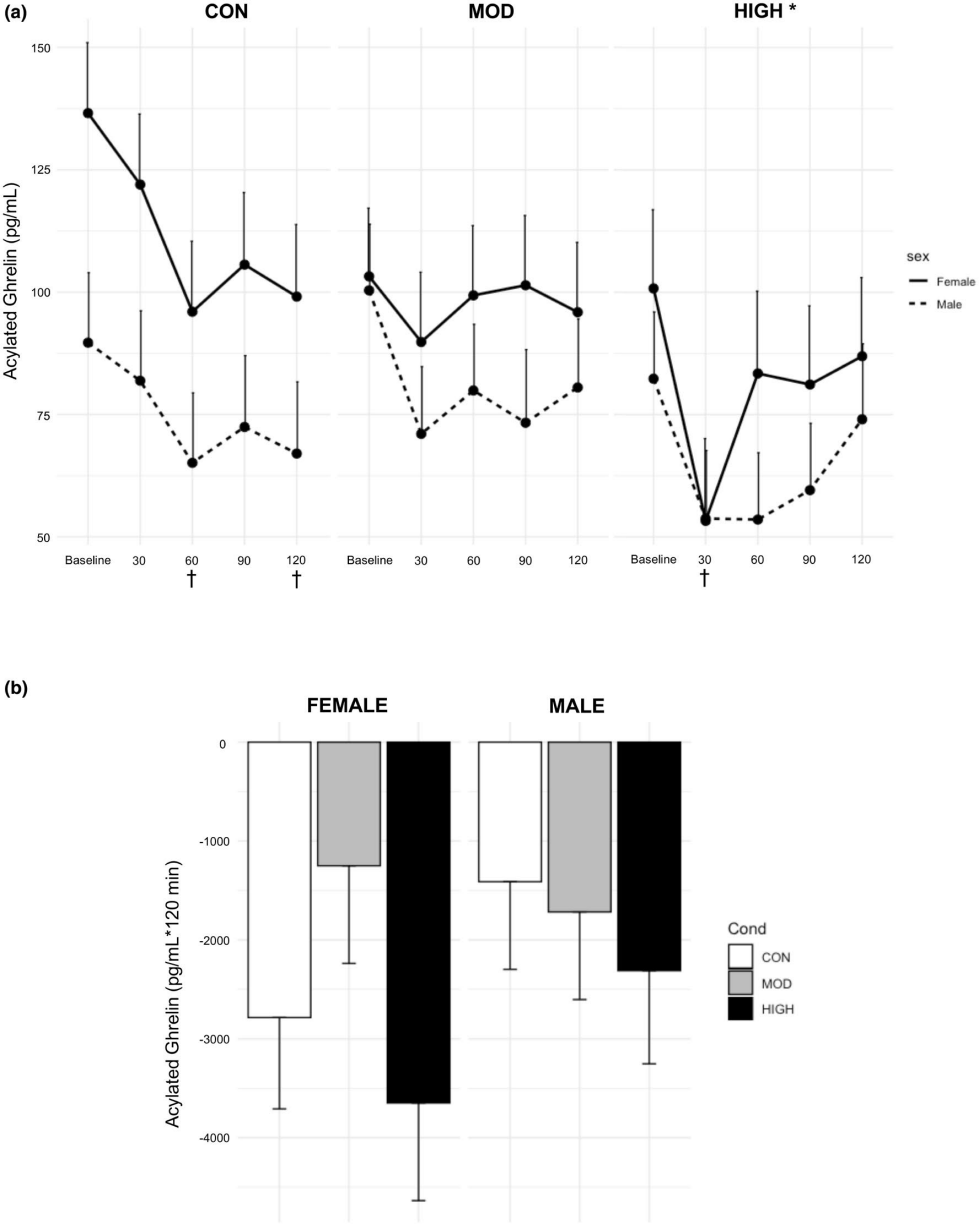
Effect of Condition on Acylated Ghrelin Levels with (a) Timepoint data and (b) Incremental AUC data. Data are from estimated marginal means of the linear mixed model and are presented as mean ± S.E. *; significantly different than CON, *p* = 0.01 †; significantly different from each condition's baseline value, *p* ≤ 0.007.

There were significant main effects for condition (*p* = 0.015, Figure 5), time (*p* < 0.0001), and sex (*p* = 0.02). There was also a significant interaction between condition and time (*p* = 0.004, Figure 5). AG levels in HIGH (72.9 ± 11.5 pg/mL) were significantly lower compared to CON (93.5 ± 11.3 pg/mL, *p* = 0.01). There was a trending difference between HIGH and MOD (89.5 ± 11.1 pg/mL, *p* = 0.07). For time, AG was decreased at all timepoints compared to baseline (*p* < 0.0001–0.004). AG levels were higher in females (97.0 ± 11.7 pg/mL) compared to males (73.6 ± 11.6 pg/mL, *p* = 0.02). AG levels in HIGH were significantly suppressed at 30 min (53.5 ± 12.7 pg/mL) compared to baseline (91.6 ± 12.4 pg/mL; *p* < 0.0001). AG levels in CON were significantly elevated at baseline (113.1 ± 12.2 pg/mL) compared to 60 (80.6 ± 12.2 pg/mL, *p* = 0.007) and 120 min (83.1 ± 12.4 pg/mL, *p* = 0.006). AG levels in HIGH at 30 min were significantly lower compared to the same timepoint in MOD (80.4 ± 11.9 pg/mL, *p* = 0.02) and CON (101.9 ± 12.2 pg/mL, *p* = 0.0002). There were no significant main or interaction effects within the AUC model (*p* = 0.23–0.47, Figure 5).

### 4.4 Deacylated ghrelin

**FIGURE 6:**
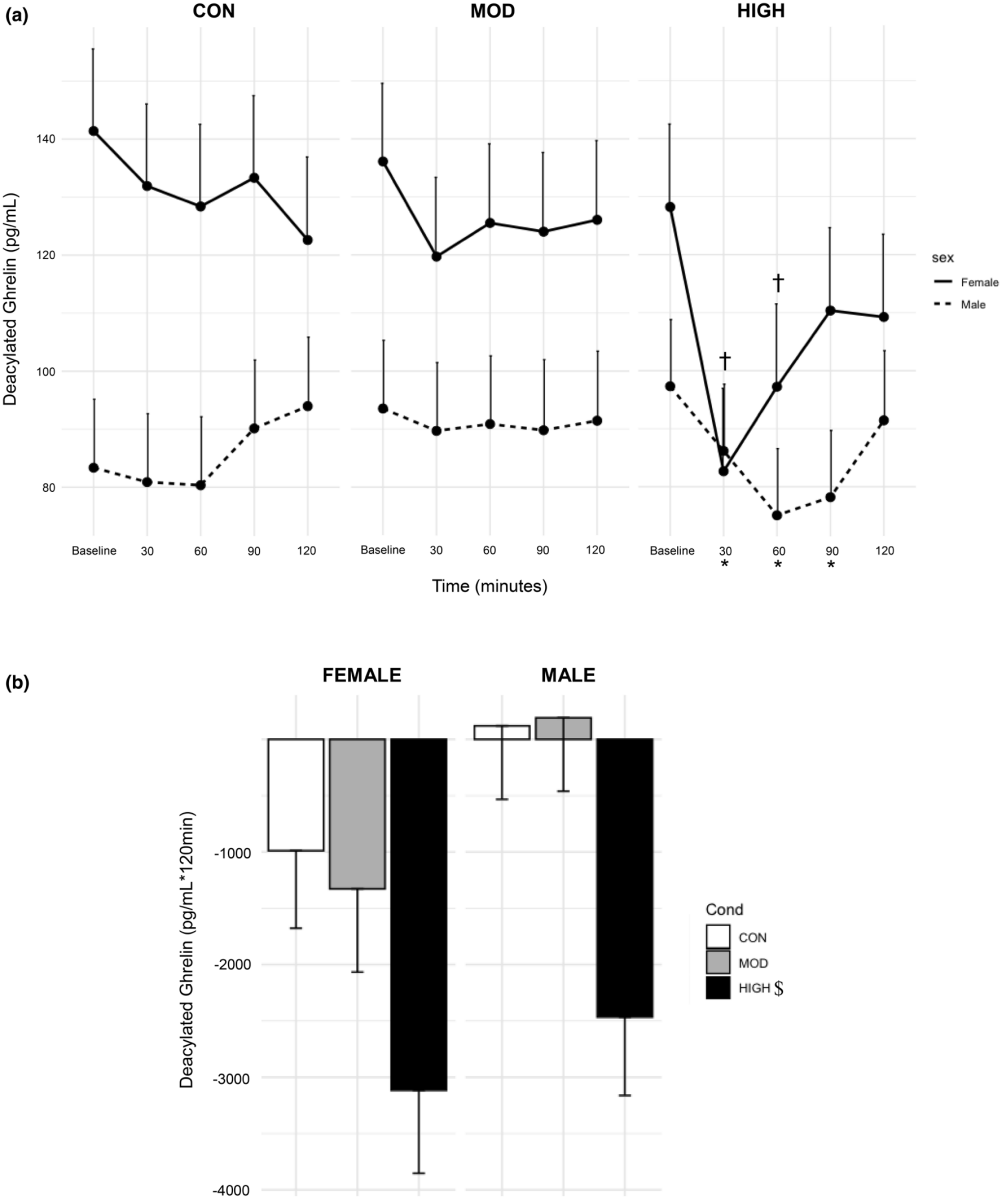
Effect of Condition on Deacylated Ghrelin Levels with (a) Timepoint data and (b) Incremental AUC data. Data are from estimated marginal means of the linear mixed model and are presented as mean ± SE. *; significantly different than each conditions baseline value, *p* ≤ 0.005 †; significantly different from each female's baseline value, *p* ≤ 0.003; $; significantly different from CON and MOD *p* = 0.002.

There was a significant main effect of time (*p* < 0.0001) and sex (*p* = 0.004). There were also interaction effects between condition and time (*p* = 0.01), sex and time (*p* = 0.006), and condition, sex, and time (*p* = 0.04). The main effect of condition had a statistical difference of *p* = 0.06. DAG levels were suppressed from 30 to 60 min compared to baseline (both; *p* < 0.0001); and a trending effect toward decreasing DAG levels at 120 min compared to baseline (*p* = 0.06). Females (121.1 ± 10.2 pg/mL) had elevated levels of DAG regardless of condition compared to males (87.5 ± 10.1 pg/mL, *p* = 0.0065). Post‐exercise levels of DAG were suppressed at 30 (84.4 ± 9.97 pg/mL, *p* < 0.0001), 60 (86.2 ± 9.97 pg/mL, *p* < 0.0001), and 90 min (94.3 ± 9.97 pg/mL, *p* = 0.009) compared to baseline (112.4 ± 10.2 pg/mL, Figure 6) in HIGH. Females had significantly higher levels of DAG (135.2 ± 10.5 pg/mL) at baseline compared to males (91.4 ± 10.3 pg/mL, *p* = 0.02), and DAG levels were decreased from 30 to 120 min compared to baseline (*p* < 0.0001–0.05) in females. Post exercise DAG levels were decreased at 30 (82.7 ± 14.3 pg/mL, *p* < 0.0001) and 60 min (97.2 ± 14.3 pg/mL, *p* = 0.003) in HIGH compared to baseline (128.2 ± 14.3 pg/mL, Figure 6) in females.

For the AUC model, there was a significant main effect of condition (*p* = 0.002, Figure 5b). The main effect of sex had a statistical difference of *p* = 0.06. HIGH (−2791 ± 505 pg/mL *120 min) was significantly lower than CON (−435 ± 472 pg/mL *120 min, *p* = 0.0045) and MOD (−568 ± 491 pg/mL *120 min, *p* = 0.009). Females had greater suppression of DAG (−1811 ± 425 pg/mL *120 min) regardless of condition compared to males (−719 ± 398 pg/mL *120 min), which approached statistical significance (*p* = 0.06).

## 5 CORRELATIONS

Correlations are listed in Table 2. For the total sample, all isoforms of ghrelin were negatively associated with FMD_diff_ (TG: *r* = −0.26, *p* = 0.0002; AG: = −0.21, *p* = 0.002; DAG: *r* = −0.28, *p* < 0.001). There were no significant associations with SR and TG/AG (*p* = 0.24–0.42), and there was a trending relationship between SR and DAG (*r* = −0.14, *p* = 0.056).

**TABLE 2:** Correlations between variables of the total, male, and female sample.

Total	FMD_diff_	SR	Males	FMD_diff_	SR	Females	FMD_diff_	SR
TG	−0.26***	0.07	TG	−0.17	0.11	TG	−0.40***	0.045
AG	−0.21**	0.10	AG	−0.16	0.20	AG	−0.27**	0.03
DAG	−0.28***	−0.14^†^	DAG	−0.10	−0.01	DAG	−0.43***	−0.16

*Note*: *r* values are listed in the figure. All values are *p* < 0.05.

** *p* < 0.01.

*** *p* < 0.001.

^†^ *p* = 0.056.

**FIGURE 7:**
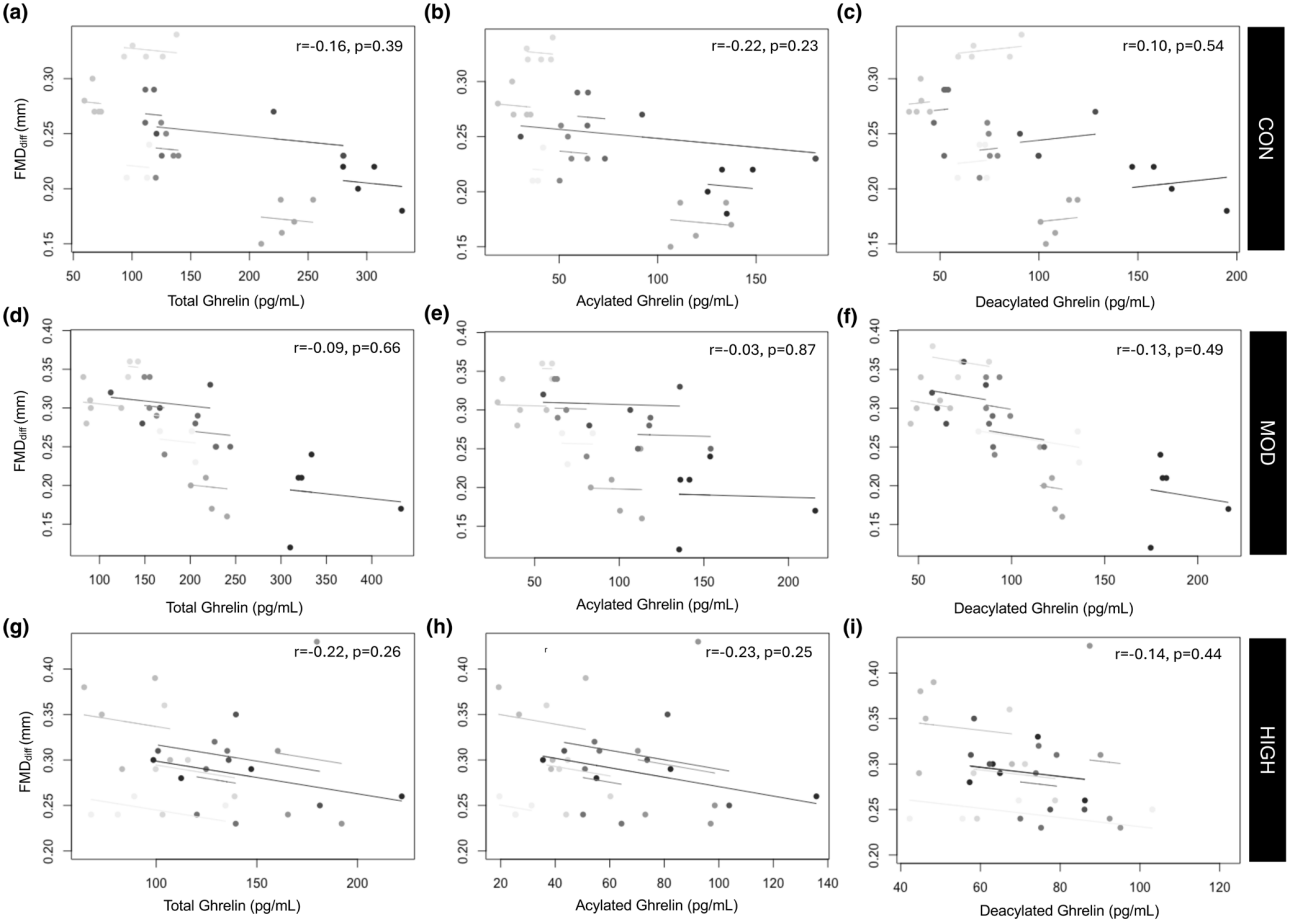
Repeated Measures Correlation Plot between FMD_diff_ and TG, AG, and DAG for the male sample for CON (a–c), MOD (D‐F), and HIGH (g–i). Each shade represents a different participant. The overall correlation represents a single correlation for all datapoints.

**FIGURE 8:**
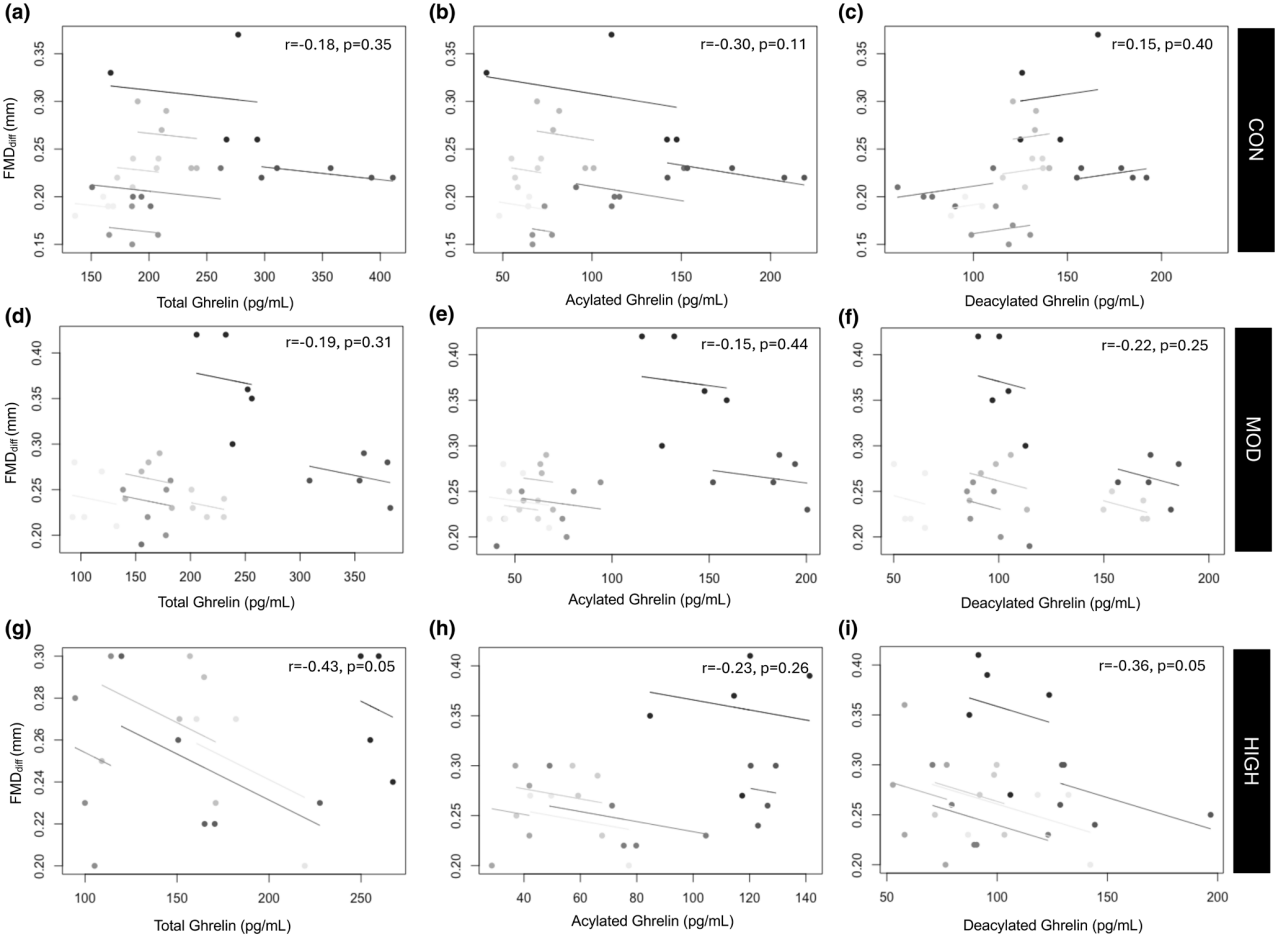
Repeated Measures Correlation Plot between FMD_diff_ and TG, AG, and DAG for the female sample for CON (a–c), MOD (D‐F), and HIGH (g–i). Each shade represents a different participant. The overall correlation represents a single correlation for all datapoints.

Within males, there was no significant relationship between ghrelin and FMD_diff_ or SR (*p* = 0.08–0.39). Within the female sample, all isoforms of ghrelin were negatively associated with FMD_diff_ (TG: *r* = −0.40, *p* = 0.0001; AG: = −0.27, *p* = 0.007; DAG: *r* = −0.43, *p* < 0.001). There were no significant associations between SR and any ghrelin isoforms (*p* = 0.12–0.78). Repeated measures correlations between ghrelin and FMD_diff_ for the female and male sample by condition are in Figures 7 and 8.

## 6 DISCUSSION

The major findings of the present study indicate that, independent of sex, isocaloric bouts of high‐ and moderate‐intensity exercise acutely augment FMD to a similar degree compared to control. Regarding ghrelin, TG, DAG, and AG levels were decreased in response to high‐intensity exercise. Further, each ghrelin isoform was negatively associated with FMD, and the relationships were stronger in females compared to males.

The effect of exercise intensity on post‐exercise FMD is equivocal, and differences in sample characteristics (i.e., age, body composition, fitness level, and health conditions) and methodology make it difficult to form a consensus (Birk et al., 2013; Hallmark et al., 2014; Jones et al., 2010). Hallmark et al. reported that only high intensity exercise improved EF; however, their sample contained younger adults than the current study, and they kept the duration constant between exercise conditions, resulting in higher caloric expenditure in their high‐intensity condition (Hallmark et al., 2014). Similarly, Weston and colleagues reported that high‐intensity interval exercise (performed both at 75% and 90% of VO_2max_) increased brachial artery FMD for up to 3 h, whereas moderate exercise had no effect (Weston et al., 2022). In contrast, studies investigating isocaloric bouts of exercise have reported similar results to the present study, with no difference in FMD between moderate and high‐intensity exercise (Johnson et al., 2012; Malin et al., 2016; McClean et al., 2015). One study found a significant difference in their %FMD data between exercise intensities when normalized for SR, but not when SR was included in the calculation (McClean et al., 2015). As the relationship between FMD and SR in our sample was weak (*r* = 0.07, *p* = 0.31), we did not normalize our FMD measurements to SR (Atkinson et al., 2009). In addition, we found that MOD led to a significant increase for up to 60 min post‐exercise, while the increase in HIGH only lasted 30 min (Figure 2). Data on the impact of exercise intensity on the post exercise time course of FMD is scarce, as few studies have examined FMD for >1 h post exercise (Dawson et al., 2013). Although not measured in the present study, it is possible that the lack of difference in FMD response was due to the inability of high‐intensity exercise to further increase bioavailable NO. In support of the above, we recently reported no differences in FMD after acute moderate or high intensity in postmenopausal females provided a placebo supplement. However, when bioavailable NO was increased via supplementation with inorganic nitrate in the form of red beetroot juice, both moderate and high‐intensity exercise significantly increased FMD compared to placebo, with high‐intensity exercise + red beet juice resulting in the greatest improvement in post‐exercise FMD (Hogwood et al., 2023).

We did not find any significant changes to SR in response to either exercise bout. Literature examining SR in response to acute exercise has differed widely, reporting an increase, decrease, or no changes (Dawson et al., 2013). A similar study investigating the effect of exercise intensity on EF also found no difference in post‐exercise SR, but they did not explore sex differences (Weston et al., 2022), while we found that females had an overall larger SR than males in our timepoint data. Our findings are reinforced by another study that measured femoral artery SR during exercise and also found that males exhibited a lower SR than females (Gonzales et al., 2009). In contrast, females had a smaller resting diameter than males, suggesting a sex difference in shear stress, an important stimulus for healthy vascular adaptations. Possible explanations include the protective effect of estrogen, increased NO bioavailability, and an enhanced antioxidant capacity found in females (Forte et al., 1998; Kander et al., 2017; Robert, 2023).

The finding that ghrelin is suppressed post‐exercise is supported by our previously published meta‐analysis (Anderson et al., 2021) and our recent study regarding exercise intensity, ghrelin levels, and appetite (Anderson et al., 2024), where the appetite study includes a subset of participants from the present study. Most of the other published studies applied a moderate‐intensity exercise stimulus and measured only AG (Anderson et al., 2021). We found that AG and DAG were decreased only after high‐intensity exercise in the present study (Figures 5 and 6). As our protocol utilized blood lactate to determine exercise intensities, these findings suggest that exercise above the lactate threshold may be necessary to elicit a suppression in ghrelin. Prior work has shown highly enriched lactate receptors within the gastric fundus (Thijssen et al., 2019) and the ability of these receptors to block ghrelin secretion from the cells within the stomach via g‐coupled receptor GPR81 (Atkinson & Batterham, 2013). It is important to note that although both TG and AG have been shown to be reduced following lactate‐mediated GPR81 signaling (Atkinson & Batterham, 2013), which may suggest a reduction in acylation, more work is needed to explore the activity of GOAT within this mechanism. However, lactate may not be the primary driver, as we previously reported moderate or non‐significant correlations between lactate and all ghrelin isoforms (Anderson et al., 2024). Additionally, in the present study, TG and AG in the control condition decreased over time. While we did not provide a morning meal, there is data showing ghrelin levels are entrained to follow the mealtime rhythm, decreasing after breakfast time regardless of if food is consumed (Natalucci et al., 2005).

We also found that there were higher levels of DAG and TG at baseline in females compared to males, and DAG levels were suppressed after high‐intensity exercise only in females in our timepoint data. Our findings suggest there may be sex differences in ghrelin release and/or degradation. A sexual dimorphism has been previously reported regarding ghrelin levels, with females having higher DAG and AG levels than males, regardless of obesity status (Broom et al., 2009; Burns et al., 2007). We report a sex difference despite similar BMI and accounting for age in our statistical models. Studies have shown that estrogen can alter circulating ghrelin levels; however, results are conflicting. Estrogen has been shown to either upregulate ghrelin levels, decrease TG levels in post‐menopausal women undergoing estrogen replacement therapy, or decrease AG following ovariectomy in rats (Rognmo et al., 2008). This sex difference was observed despite differences in body composition, as females had elevated BF% compared to males, and increased adiposity is associated with reduced TG and DAG (Rodríguez et al., 2009). Collectively, these results strengthen the need for more work examining sex differences within gut hormones.

Additionally, we observed some statistically significant relationships between EF and ghrelin levels (Table 2). Studies in humans (Iantorno et al., 2007; Kleinz et al., 2006; Tesauro et al., 2009) and animals (Iantorno et al., 2007; Shimizu et al., 2003) have identified ghrelin as a vasodilator, likely through NO‐mediated mechanisms (Iantorno et al., 2007). However, most studies that have illustrated a significant effect on the vasculature have applied a supraphysiological dose of ghrelin (often directly treating cells), whereas the present study examined the physiological response of endogenous levels of ghrelin to exercise of differing intensity. Interestingly, we present negative associations between ghrelin levels and FMD; however, the strength of the relationship was weak in males and only moderate in females (Figures 7 and 8). This may be due to the complicated physiology of endocrine hormones, where the target functions can be down‐ or upregulated in response to hormone levels. Supporting this, prior data has identified ghrelin in secretory vesicles of the cytoplasm of endothelial cells, and the synthesis of ghrelin may be modulated by changes in circulating ghrelin levels. These changes then act through endothelial cell receptors, possibly through a negative feedback loop; providing a possible explanation for the direction of our correlations (Kleinz et al., 2006). We also report that DAG levels were suppressed post‐exercise in females only, and statistically significant relationships between FMD and ghrelin were only seen in females. Although FMD was augmented in both males and females, the pathways mediating such changes may differ by sex and are likely multifactorial. However, as FMD was increased in response to both exercise intensities, and ghrelin was suppressed following only high intensity, clearly ghrelin and its isoforms are not the primary driver of the augmentation of EF after acute exercise.

There are several limitations of this study. Repeated measures correlations assume the same slope for each subject, which may have impacted the results. As our protocol included an overnight fast for each visit, real‐world application is weakened as most individuals consume mixed meals before and/or after exercise. Therefore, future work should examine the impact of meal content on each ghrelin isoform and EF in response to exercise. In addition, the differences in fitness levels and exercise caloric expenditure between males and females may have impacted our results. Finally, we only tested lean subjects without evidence of endothelial dysfunction. As the ghrelin/GOAT axis and EF are dysregulated in obesity (Iantorno et al., 2007), results of the present study may not apply to individuals with overweight or obesity or other conditions that impact vascular health.

In conclusion, we report that exercise augments FMD independent of sex, and ghrelin is transiently suppressed after high‐intensity exercise. In addition, the present data suggest that exercise‐induced improvement in FMD is only weakly to moderately related to changes in ghrelin levels, and sex and ghrelin isoform may impact this relationship. More work is needed to examine the differential FMD and ghrelin responses to exercise of differing exercise doses and determine if adiposity further impacts this response.

## AUTHOR CONTRIBUTIONS

KCA, JDA, and AW conceived and designed research; KCA, EEG, BS, MES, NRW, ZL, and KML collected data; KCA and EEG analyzed data; KCA interpreted results, prepared figures, and drafted manuscript; all authors edited and revised the manuscript and approved the final version.

## FUNDING INFORMATION

UVA School of Education and Human Development, NIDDK (5R01DK129510–02; 5T32DK007646).

## CONFLICT OF INTEREST STATEMENT

The authors have nothing to disclose.

## Data Availability

Some or all datasets generated during and/or analyzed during the current study are not publicly available but are available from the corresponding author on reasonable request.
